# A conserved Delftibactin A biosynthetic gene cluster plays an important role in the biocontrol of kiwifruit bacterial canker by *Delftia lacustris* ZWP15

**DOI:** 10.1128/spectrum.00313-26

**Published:** 2026-05-29

**Authors:** Jing Huang, Weipeng He, Yifei Liang, Xuyan Wu, Ruolan Yang, Xinying Liu, Zimeng Wang, Nana Wang, Lili Huang

**Affiliations:** 1 College of Life Sciences, State Key Laboratory for Crop Stress Resistance and High-Efficiency Production, Northwest A&F University 213667, Yangling, Shaanxi, China; 2 College of Plant Protection, State Key Laboratory for Crop Stress Resistance and High-Efficiency Production, Northwest A&F University 546345, Yangling, Shaanxi, China; USDA-ARS San Joaquin Valley Agricultural Sciences Center, Parlier, California, USA

**Keywords:** kiwifruit bacterial canker, *Delftia lacustris*, biosynthetic gene cluster, *Pseudomonas syringae *pv. *actinidiae*, antimicrobial metabolites

## Abstract

Kiwifruit bacterial canker (KBC), caused by *Pseudomonas syringae* pv. *actinidiae* (*Psa*), is a globally devastating disease with limited sustainable control options. Here, we report *Delftia lacustris* strain ZWP15 as a novel biocontrol agent exhibiting strong antagonism against *Psa in vitro* and in field trials. Comparative genomics identified a conserved Delftibactin A biosynthetic gene cluster in ZWP15, which was functionally validated through gene knockout. Loss of the Delftibactin A biosynthetic gene cluster significantly impaired ZWP15’s antibacterial activity, while crude extracellular extracts from wild-type ZWP15 markedly inhibited *Psa* growth and suppressed key virulence traits, including motility, biofilm formation, and transcription of type III secretion system genes. Importantly, these extracts reduced *Psa* colonization and disease severity in kiwifruit plants, suggesting a dual role of Delftibactin A-associated metabolites in direct pathogen inhibition and virulence disruption. To our knowledge, this is the first demonstration of *D. lacustris* as a biocontrol agent against KBC. Our findings highlight the potential of exploiting the Delftibactin A biosynthetic gene cluster for developing sustainable alternatives to chemical bactericides.

**IMPORTANCE:**

Kiwifruit bacterial canker (KBC) is a devastating disease with scarce sustainable management options. This study identifies *Delftia lacustris* as a novel and effective biocontrol agent against KBC. The key molecular mechanism underlying the antibacterial activity of *Delftia lacustris* ZWP15 is the production of Delftibactin A. Besides the direct antibacterial effect, Delftibactin A also uniquely disrupts key pathogenic pathways of the pathogen, including motility, biofilm formation, and the type III secretion system. This dual mode of action enhances its potential for sustained control of the disease. This study highlights the promising prospects of developing the Delftibactin A biosynthetic gene cluster into an environmentally friendly alternative to traditional copper-based bactericides.

## INTRODUCTION

Kiwifruit bacterial canker (KBC), caused by *Pseudomonas syringae* pv. *actinidiae* (*Psa*), is a globally devastating disease affecting all major kiwifruit cultivars (*Actinidia* spp.) (1). KBC can infect the plant at different growth periods throughout the year and leads to kiwifruit leaf spots, flower rot, tree branch canker, necrosis of branches or trunk, and can even destroy entire orchards (2, 3). It has been reported that the pathogen can persist asymptomatically in host tissues for over a year, complicating early detection and control (4). Current management relies mainly on copper-based bactericides and surgical removal of infected tissues (5). However, excessive chemical use has led to widespread *Psa* resistance and environmental contamination (6, 7), underscoring the urgent need for sustainable alternatives.

Biological control has gained increasing attention due to its environmentally friendly, nontoxic characteristics and efficiency (8). In recent years, antagonistic microorganisms have been considered one of the most promising green and sustainable approaches for plant disease control, and several species have been assessed for their potential in the biological control of KBC. For example, *Bacillus cereus* B2 isolated from soil showed antibacterial activity to *Psa* under laboratory and field conditions (9). *Pseudomonas bijieensis* strain XL17 showed good control effects on KBC by producing 2,4-diacetyl phloroglucinol and lipopeptides (10). *Paenibacillus polymyxa* YLC1, obtained from kiwifruit rhizosphere soil, showed potent suppression of KBC *in vitro* and field evaluations mediated by polymyxin B1 (11). However, most of the existing reports focus on a few strains of *Bacillus* sp. (9) and *Pseudomonas* sp. (10, 12), and their application potential and environmental adaptability are limited due to the differences in strain specificity. Thus, expanding the diversity of biocontrol resources remains a critical challenge.

Although there are only a few reports on the application of *Delftia* sp. in plant disease management, they have demonstrated significant potential in this field. For instance, the strain *Delftia lacustris* PPO-1 as a biocontrol bacterium directly inhibited the mycelial growth of fungal pathogens *in vitro* and significantly reduced disease incidence in tomatoes (13). *Delftia tsuruhatensis* MTQ3, isolated from the tobacco rhizosphere, exhibited antagonistic effects against both *Ralstonia solanacearum* and *Phytophthora nicotianae* (14). In addition, *Delftia* sp. D-75, which can produce Delftibactin A, has been reported to be active against *Acinetobacter baumannii*, *Klebsiella pneumoniae*, and *Pseudomonas aeruginosa* (15). Notably, the Delftibactin A biosynthetic gene cluster is commonly present across the *Delftia* spp. and has been employed as a molecular marker for identifying *Delftia* spp. in environmental samples (16). However, *Delftia* sp. has not been reported as a biocontrol strain for KBC.

In this study, we report for the first time that a *Delftia lacustris* strain ZWP15, which was isolated from kiwifruit rhizosphere soil, is effective against KBC in isolated branches, isolated leaf discs, and under field conditions. Comparative genomics analysis revealed that, while the genomes of *Delftia* sp. are characterized by plasticity and diversity in gene composition and family counts, the Delftibactin A gene cluster remains remarkably conserved. The knockout of key genes within the Delftibactin A gene cluster revealed their significant role in the biological control activity of *D. lacustris* ZWP15. Additionally, the crude extract of Delftibactin A not only modulates *Psa* virulence traits by reducing motility, impairing biofilm formation, and suppressing expression of pathogenicity-related genes associated with the type III secretion system (T3SS) but also inhibits *Psa* infection and the subsequent destruction of host cells. This study addresses the existing gap regarding the potential of *Delftia* sp. in the prevention and treatment of KBC and provides a theoretical foundation for the development of broad-spectrum biocontrol agents based on *Delftia* sp. and the engineering of optimized strains. Moreover, the diversity of *Delftia* sp. and the conservation of the Delftibactin A cluster suggest a robust environmental adaptability, which is anticipated to enhance the stability of biocontrol agents in complex field environments.

## MATERIALS AND METHODS

### Bacterial strains and culture conditions

*Psa* M228 was isolated from *Actinidia chinensis* var. *chinensis* “Hongyang” in our previous study (17) and was used to assess the antibacterial activity of the strains. *Psa* M228 were cultured in Luria Broth (LB) medium. *Psa* M228-GFP, constructed in our previous study (4), was plated on LB medium containing 50 μg/mL kanamycin. After an assessment of the antagonistic effects of fermentation broths obtained from ZWP15 cultured in potato dextrose broth (PDB) and LB liquid media, the PDB with superior efficacy was selected for further cultivation (Fig. S1). Additionally, potato dextrose agar (PDA) was adopted for plate-based assays. All strains were cultured at 28°C for 48 h at 220 rpm for further study. All the strains were identified and preserved in the State Key Laboratory for Crop Stress Resistance and High-Efficiency Production, Northwest A&F University, Yangling, China.

### Isolation of antagonistic bacteria

To identify potential biocontrol bacteria against *Psa*, rhizosphere soil was collected from asymptomatic kiwifruit trees located in orchards experiencing severe KBC epidemics across various locations (18). To isolate antagonistic microorganisms, rhizosphere soil (5 g) was added to sterile water (100 mL) and shaken at 220 rpm for 10 min at 20°C–30°C. The resulting suspension was then diluted 100-fold, and aliquots were plated on solid LB plates. Single colonies were subsequently collected for antibacterial activity assays.

### Evaluation and identification of antagonistic bacteria

For the antibacterial activity assays, mixed plates were prepared by mixing 1 mL of *Psa* bacterial suspension (10^9^ CFU mL^−1^) with 100 mL of solid LB medium. The preserved microorganisms were inoculated separately onto these mixed-media plates using sterile toothpicks. The antibacterial diameters were measured after culturing at 28°C for 72 h. All strains underwent two rounds of subculturing to ensure purity and were then preserved in 30% glycerol at −80°C for future use.

The strain exhibiting the strongest antifungal activity in the modified dual culture assays, designated as ZWP15, was further identified based on morphological and phylogenetic methods. The ZWP15 strain was cultured on a PDA agar plate at 28°C, and its growth rate, colony morphology, and color were observed. Genomic DNA was extracted from the colony using the Bacterial DNA Kit (Omega Bio-Tek, Guangzhou, China). Fragments of the 16S rDNA and *gyrB* genes were amplified using the primers 27F (5′-AGAGTTTGATCMTGGCTCAG-3′) and 1492R (5′-GGTTACCTTGTTACGACTT-3′) for 16S rDNA, and the primers *gyrB*-F (5′-CCNGGNATGTAYATHGG-3′) and *gyrB*-R (5′-CATYTCNCCNARNCCYTTRWANCKKTG-3′) for *gyrB*. The resulting products were purified and sequenced by Sangon Biotech Co., Ltd. (Shanghai, China). Reference sequences were downloaded from the NCBI database (https://www.ncbi.nlm.nih.gov/) based on BLASTn search results. The 16S rDNA and gyrB gene sequences were concatenated and aligned using MEGA11 with the ClustalW algorithm under default parameters. Phylogenetic trees were constructed using the neighbor-joining method (19) with the Kimura 2-parameter model, and bootstrap analysis was conducted with 1,000 replicates.

### Testing biocontrol effects of antagonistic bacteria in the greenhouse

Two-year-old susceptible varieties of kiwifruit trees, *Actinidia chinensis* var. *chinensis* “Hongyang,” were adopted to evaluate the biocontrol effect of bacterial strains on KBC *in vitro* by leaf-disk inoculation via vacuum infiltration and branch inoculation via cutting wounds (20). Healthy leaves were punched out of the leaf discs with a 1.2 cm diameter punch and immersed in 30 mL bacterial culture (1 × 10^5^ CFU mL^−1^) in a 50 mL tube. After the vacuum infiltration treatment, the infiltrated leaf discs were placed flat on a 0.1% water agar medium and cultured in a relative humidity of 95% for 24 h (16 h in the light at 10°C and 8 h in the dark at 4°C), and the lesion size was measured after 4 days. Healthy branches were cut into 15-cm-long twigs, and the ends were sealed with molten paraffin according to the previous method (17). Ten microliters of bacterial culture (2 × 10^6^ CFU mL^−1^) was dripped onto the wound, which was cut into the xylem of the twigs using blades. The inoculated twigs were kept in the artificial climate chamber above. The lesion length was measured after 20 days. Water served as a negative control.

### Testing biocontrol effects of antagonistic bacteria in the field

Field experiments were carried out at three different kiwifruit orchards planted with *A. chinensis* var. *deliciosa* “Cuixiang” (8-year-old) and *A. chinensis* var. *deliciosa* “Jinfu” (12-year-old) in Xieshang, Yangling, Shaanxi (34°2958 563N 106°4350 563E) in China (4). The kiwi trees were sprayed with the fermentation broth of the bacterial strain (3 × 10^8^ CFU mL^−1^) twice during the period from harvest to leaf fall (from October to November), with a spraying interval of 12 days. The biocontrol effect was assessed in March of the following year. Zhongshengmycin (Fujian Kaili Biological Products Co., Ltd, China), kasugamycin (Wuhan Kono Biotechnology Co., Ltd, China), and *Bacillus subtilis* powder (Yangling Lvdu Biotechnology Co., Ltd, China) were used as positive controls according to the instruction manual, as they represent commonly used commercial products for KBC management in agricultural practice. Control effect was calculated as: [(incidence in control − incidence in treatment)/incidence in control] × 100%. Data are calculated as mean values ± standard deviation (*n* = 9, three orchards with three replicates each). Statistical significance (*P* < 0.05) was determined by one-way ANOVA followed by Student’s test, with different superscript letters indicating significant differences among treatments.

### Comparative genomic analysis of *D. lacustris* ZWP15

Genome sequencing of ZWP15 was carried out by Personal Biotechnology Company (Shanghai, China) using a hybrid sequencing strategy by the Nanopore PromethION48 platform (Pacific Biosciences) and the Illumina NovaSeq platform. Raw data obtained by Nanopore platform sequencing were assembled by Flye (21) and Unicycler (22) software, followed by the integration of hybrid assemblies to construct a contiguous genome. The genome sequence was refined to completion via pilon software (23), ensuring nucleotide accuracy and contiguity.

Based on the phylogenomic framework of a previous study (16), which divides the genus *Delftia* into two major lineages (the “*Delftia acidovorans”* clade and the “*D. lacustris* and *D. tsuruhatensis”* clade), six additional *Delftia* spp. genomes were selected for comparative analysis. *Delftia deserti* was included as it is the only other *Delftia* species with a completed and publicly available genome in NCBI beyond the three common species. The genomes of six additional *Delftia* spp. were downloaded from the NCBI database. A circular comparative genomic map was constructed using BRIG (24), with *D. lacustris* strain ZWP15 serving as the reference genome alongside the six *Delftia* spp. The upset map depends on the Chiplot online platform (https://www.chiplot.online/) (25). The microbial secondary metabolite synthesis gene clusters of seven *Delftia* spp. were predicted using antiSMASH software (26). The completion of the comparison gene cluster map similarly depends on the Chiplot online platform (25).

### Deletion of genes encoding antimicrobial activity using PCR

Based on the gene cluster information related to the active substances analyzed above, a complete biosynthetic gene cluster for Delftibactin A was identified. To elucidate the contribution of Delftibactin A in the antagonistic activity of *D. lacustris* ZWP15, two deletion mutants, Δ*tycC* and Δ*bacA,* were constructed using the homologous recombination method employing pK18mobSacB (27, 28). The deletion vectors were constructed by ligating the left and right arms of the target genes to pK18mobSacB and introducing it into *D. lacustris* ZWP15 by conjugal mating experiments (28). Gene deletion efficacy was validated by PCR and sequencing (Fig. S2). The corresponding primers for the target gene amplification and PCR-detected primers are listed in Table S1.

### Preparation of crude extract of active substances

The fermentation broth (whole culture broth, including both bacteria and supernatant) of *D. lacustris* ZWP15 and its mutants, Δ*tycC* and Δ*bacA,* were collected after single clones were inoculated and cultured in PDB at 28°C and 220 rpm for 72 h. The active compounds were extracted from fermentation broth by adding an equal volume of ethyl acetate (1:1, vol/vol). The organic phase was then evaporated using a rotary evaporator, and the resulting extract was reconstituted in methanol. The activity of the crude extracts was subsequently evaluated (Fig. S3).

### Effect of the active crude extract on swimming and swarming motility of *Psa*

*Psa* M228 was cultured at 28°C and 220 rpm in LB medium until the logarithmic growth phase. The bacterial cells were collected by centrifugation at 4,000 rpm for 2 min and resuspended in sterile water to a final concentration of 10^8^ CFU/mL. The LB plate containing 0.25% or 0.4% agar, with or without active extracts from strain ZWP15 and its mutants, Δ*tycC* and Δ*bacA*, was used to assess the motility of *Psa*. For swimming motility, 1.5 μL of standardized bacterial suspension was spotted at the center of an LB plate containing 0.25% agar, and the plates were incubated face up at 28°C for 15 h. For swarming motility, 1.5 μL of standardized bacterial suspension was spotted at the center of an LB plate containing 0.4% agar, and the plates were incubated face up at 28°C for 15 h. After incubation, the diameter of the motility zone was measured, and the plate was photographed (29).

### Bacterial cell morphological observation by scanning electron microscope

The microscopic features of *D. lacustris* ZWP15 were observed using a scanning electron microscope (SEM). A cover glass (≤7 mm) with a drop of water was placed in a high concentration of *D. lacustris* ZWP15 bacterial solution (OD_600_ = 1.0) for 24 h. The cover glass adhered with *D. lacustris* ZWP15 was fixed by soaking in 2.5% glutaraldehyde at 4°C for 2 h. Then, the glass slides were fixed with osmium acid (0.7 mL, 1% [vol/vol]) at 4°C for 1–2 h after being washed two to three times with 0.1 mol L^−1^ PBS buffer (pH 7.2), each time for 10 min. The glass slide samples were dehydrated through a graded ethanol series (30%, 50%, 70%, 80%, and 90%), followed by two changes of 100% ethanol, each time for 10 min, and replaced once with 1 mL of isoamyl acetate for 10 min. Finally, samples were critical-point dried with carbon dioxide, gold-sputtered, and then observed and photographed by SEM (Nova Nano SEM 450, USA) at 15 kV.

### Effect of the active crude extract on biofilm formation of *Psa*

Biofilm assays of *Psa* M228 were first observed by SEM. The cover glass, after the addition of a drop of water, was left to stand in *Psa* M228 bacterial solution (OD_600_ = 1.0), which was treated in advance with crude extracts of ZWP15 for 24 h. The next steps were the same as Bacterial cell morphological observation by scanning electron microscope.

In addition to SEM observations, the effect of crude extract on the biofilm formation of *Psa* was also assessed using the previously reported method (30). In a sterile 96-well tissue culture plate, 200 µL of *Psa* bacterial suspension and ZWP15 crude extract (at concentrations of 0, 0.01, 0.02, 0.03, 0.04, 0.05, 0.075, and 0.1 mg mL⁻¹, respectively) were added. The plate was incubated at 28°C for 24 h. Subsequently, the contents of each well were aspirated and washed three times with 250 µL of sterile water. After being fixed with 200 µL of 99% methanol for 15 min, the contents in each well were aspirated and inverted to dry. Then, 200 µL of 2% crystal violet was used for staining for 5 min, and the contents of each well were aspirated and washed three times with 250 µL of sterile water. Once the plate was completely dried, the adhered dye was dissolved with 160 µL of 33% (vol/vol) glacial acetic acid. Absorbance (OD_570_) was measured using a Thermo Multiskan EX Microplate Photometer (Thermo Fisher Scientific Inc., USA), both before and after the addition of glacial acetic acid. The difference in OD_570_ values indicates the biofilm-forming ability of *Psa*.

### Effect of the active crude extract on the expression of T3SS-related genes of *Psa*

T3SS is a key pathogenic factor in *Psa*. To assess the impact of the active crude extract on the expression levels of T3SS-related genes, including the transcriptional regulators *avrE1*, *hopR1*, and *hrpL* (17), the following experimental procedure was employed. The active crude extract from strain ZWP15 (0.5 mg mL^−1^) was added to *Psa* bacterial suspension precultured to the logarithmic phase and fermented at 28°C and 220 rpm for 6 or 12 h. Bacteria were collected by centrifugation at 4°C and 10,000*g* for 10 min. Total RNA was extracted from bacterial cultures using the RNA pure Bacteria Kit (Jiangsu CoWin Biotech Co., Ltd., China), and reverse-transcribed into cDNA using the Revert Aid First Strand cDNA Synthesis Kit (Thermo Scientific, MA, USA). RT-qPCR was performed on a LightCycler 480 System with ChamQ SYBR qPCR Master Mix (Vazyme, Q311, Nanjing, China). The *gyrB* gene was used as the endogenous control. Relative expression levels of the target genes (*avrE1*, *hopR1*, and *hrpL*) were calculated using the 2^-ΔΔCt^ method (31). All reactions were performed in triplicate, with three independent biological replicates to ensure accuracy.

### Inhibition of the active crude extract on the expansion of *Psa* M228 in kiwifruit leaves

Leaves of *Actinidia chinensis* var. *chinensis* “Hongyang” were puncture-inoculated in the main vein at 1 cm from the petiole using a sterile syringe. The treatment group was pretreated with the crude extract of ZWP15, while the control group was pretreated with sterile water. After 24 h, the puncture sites in both groups were inoculated with *Psa* M228-GFP. All inoculated leaves were subsequently placed in a humidified incubator at 16°C for 5 days, after which the expansion distance was recorded.

The leaf vein tissues 1 cm above the puncture sites were excised, surface-sterilized with 75% ethanol for 30 s, and rinsed three times with sterile water. The tissues were homogenized in PBS buffer, and the final volume was adjusted to 1 mL. Serial twofold dilutions were prepared, and 10 μL of each dilution was plated on LB plates containing 50 μg/mL kanamycin (Fig. S4). The single colonies were counted after culturing at 28°C for 48 h.

### Cytological observation of kiwifruit tissues by transmission electron microscope

Sample preparation for transmission electron microscope (TEM) observation was performed following established protocols with minor modifications (12). Briefly, leaf disc samples were minced and fixed overnight in 4% glutaraldehyde in 0.1 mol L^−1^ PBS buffer (pH 6.8) at 4°C and then in 1% osmium tetroxide for 1.5 h. After that, leaf disc samples were dehydrated sequentially in 30%, 50%, 70%, 80%, 90%, and 100% ethanol, followed by sequential infiltration with White resin (London Resin Company Ltd., England). Polymerization occurred at 55°C following embedding in a pure resin medium. Semithin and ultrathin sections were prepared with a UC7 ultramicrotome (Leica Microsystems, Germany). Semithin sections were stained in 1% (wt/vol) toluidine blue and imaged with a BX53/DP70 microscope (Olympus, Japan). Ultrathin sections were plated on 100-mesh formvar-coated copper grids and double-stained with uranyl acetate and lead citrate and then examined by HT-7700 TEM (Hitachi, Japan) at 80 kV.

### Determination of the relative conductivity of kiwifruit leaves

To evaluate the structural integrity of plant cells, we measured variations in the relative conductivity of plant leaves (32). Leaf disc samples were processed following the same protocol described in Testing biocontrol effects of antagonistic bacteria in greenhouse. After infiltration, leaf discs were incubated at 95% relative humidity for 24 h (16 h light at 10°C and 8 h dark at 4°C). At each designated time point (0, 8, 16, 24, 32, 40, and 48 h), approximately 0.1 g of leaf disc samples was collected in a test tube containing 10 mL of distilled water and soaked at room temperature for 5 h (12). The conductivity R1 was determined using HACH portable meters series (HACH, USA). The samples were then boiled in a water bath for 20 min, cooled to room temperature, and then the conductivity R2 was determined. The ratio of R1 to R2 is regarded as the relative conductivity.

## RESULTS

### Identification of the antagonistic strain ZWP15 as *Delftia lacustris*

**Fig 1:**
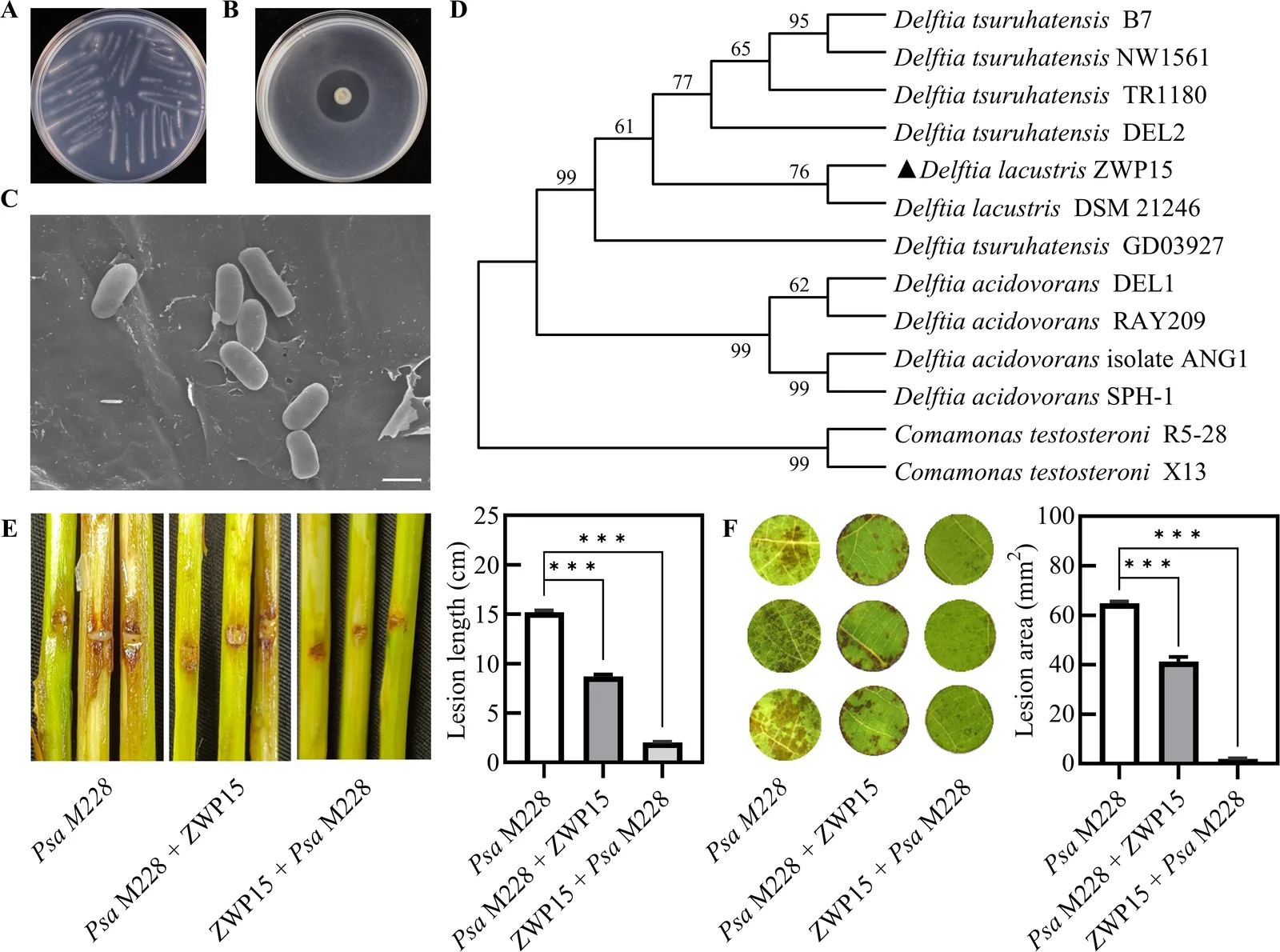
Isolation and identification of antagonistic strain ZWP15. (**A**) Colony morphology of ZWP15 cultured on PDA medium at 28°C for 48 h. (**B**) Antagonistic activity of ZWP15 against *Psa* on PDA plates, demonstrating a clear inhibition zone. (**C**) Scanning electron micrograph of ZWP15, revealing rod-shaped cellular morphology. Bar, 1 μm. (**D**) Phylogenetic analysis of ZWP15 based on concatenated 16S rDNA and *gyrB* gene sequences. (**E and F**) Biocontrol efficacy of ZWP15 against *Psa*-induced KBC on detached twigs (**E**) and leaf discs (**F**) of *Actinidia chinensis* var. *chinensis* “Hongyang” (***P < 0.001, Student’s *t*-test).

Strain ZWP15 demonstrated strong antagonistic activity, forming an inhibition zone exceeding 20 mm in diameter (Fig. 1A and B). On PDA medium, the colonies appeared white, round, and surrounded by a slightly transparent halo (Fig. 1A). SEM revealed that the cells were rod-shaped (Fig. 1C). Molecular identification was performed by sequencing the 16S rDNA and *gyrB* genes, followed by phylogenetic analysis (Fig. 1D). Based on morphological and molecular characteristics, strain ZWP15 was identified as *Delftia lacustris*.

To assess the biocontrol potential of *D. lacustris* ZWP15 against *Psa*, pathogenicity tests were conducted on detached leaves and twigs of *Actinidia chinensis* var. *chinensis* “Hongyang.” Leaves inoculated with *Psa* M228 alone developed severe browning and necrosis, with lesions covering 64.82% of the total leaf area. In contrast, leaves treated with ZWP15 before or after *Psa* inoculation showed minimal symptoms, with lesion areas reduced to only 41.23% (*Psa* M228 + ZWP15) and 1.96% (ZWP15 + *Psa* M228) (Fig. 1E). Similarly, lesion lengths were significantly shorter in ZWP15-treated samples (2.01 mm for ZWP15 + *Psa* M228) compared to *Psa* M228 alone (15.2 mm) or co-inoculated samples (8.7 mm for *Psa* M228 + ZWP15). Furthermore, pretreatment with ZWP15 (ZWP15 + *Psa* M228) effectively suppressed disease progression in detached twigs compared to the *Psa* M228 control (Fig. 1F).

To assess the field efficacy of *D. lacustris* ZWP15 in controlling KBC, disease incidence was monitored in late March of the next year. Application of a 10-fold dilution of ZWP15 fermentation broth showed strong inhibitory effects, achieving control rates of 80.97% in *Actinidia chinensis* var. *deliciosa* “Jinfu” and 81.69% in “Cuixiang.” These efficacy rates were significantly superior to both kasugamycin (71.92%) and *Bacillus subtilis* powder (72.70%) treatments (Table 1).

**TABLE 1:** Biocontrol effect of *D. lacustris* ZWP15 against KBC in the field^*a*^^,^^*b*^

Treatments	*A. chinensis* var. *deliciosa* “Jinfu”		*A. chinensis* var. *deliciosa* “Cuixiang”	
	Average of diseased branches (%)	Control effect (%)	Average of diseased branches (%)	Control effect (%)
*D. lacustris* ZWP15	1.45	80.97 ± 0.57a	1.95	81.69 ± 1.22a
Kasugamycin	2.14	71.92 ± 0.66b	2.35	77.93 ± 0.69a
*B. subtilis* powder	2.08	72.70 ± 1.16b	2.77	73.99 ± 0.28b
Control treatment (CK)	7.62	–	10.65	–

^*a*^ According to the calculation formula for control effect: [(incidence in control - incidence in treatment) / incidence in control] × 100%. The control effect (%) in the control treatment does not exist, so it is represented by “–”.

^*b*^ Values with different superscript letters (a, b) indicate statistically significant differences at the *P* < 0.05 level. Values sharing the same letter are not significantly different.

### The Delftibactin A gene cluster is highly conserved within the *Delftia* spp.

**Fig 2:**
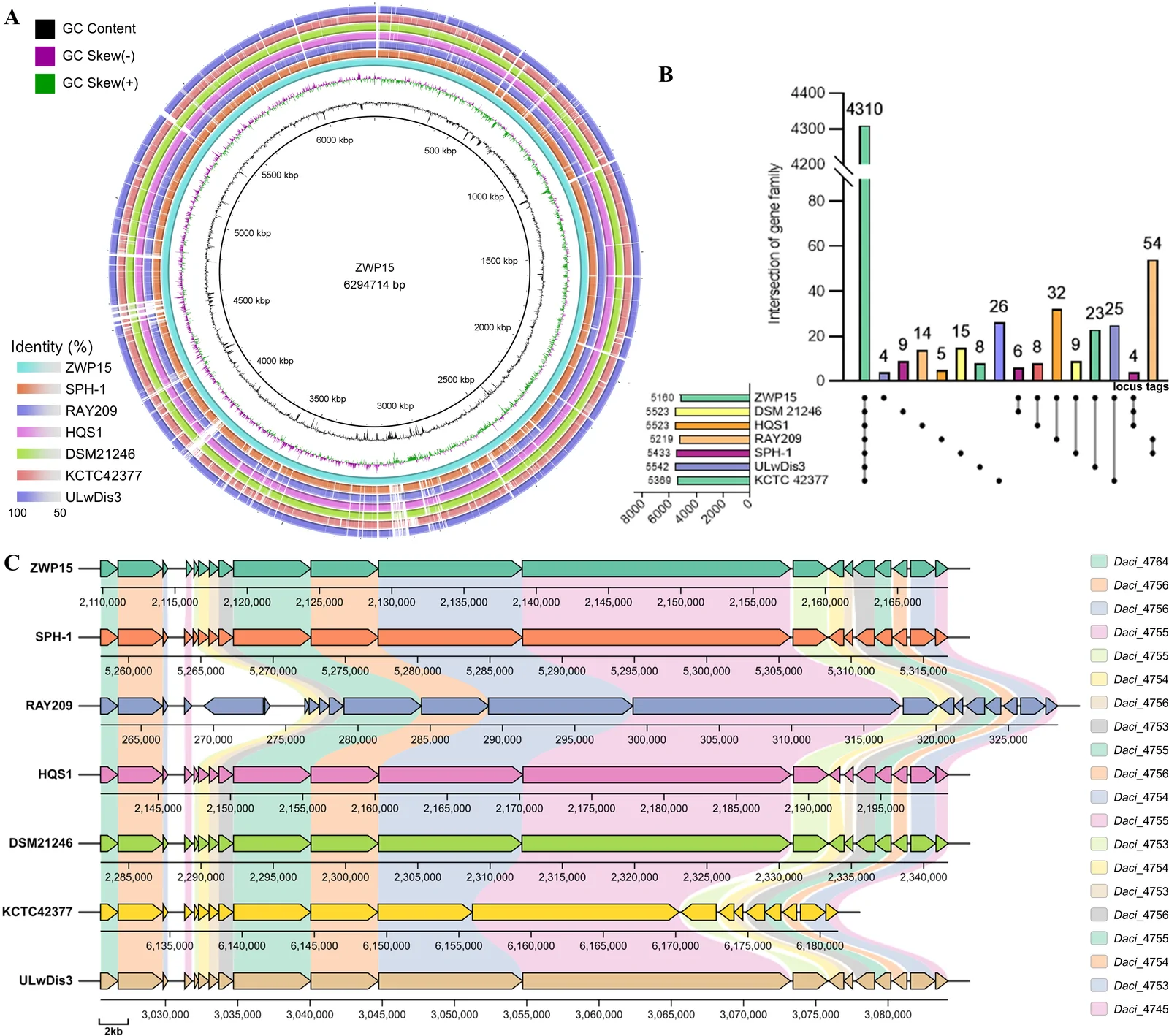
The Delftibactin A gene cluster is highly conserved within the *Delftia* spp. (**A**) Circular genome comparison of seven *Delftia* spp., showing conserved genomic features and strain-specific elements. (**B**) Orthologous gene family distribution among the seven strains, visualized using an Upset plot. The left horizontal bar chart shows the total number of gene families for each strain. In the dot plot, each individual dot represents a strain, and the corresponding column above represents the number of unique gene families of that strain. If multiple dots are connected by lines, the corresponding column above represents the number of common gene families shared by these strains. (**C**) Comparative organization of Delftibactin A biosynthetic gene clusters in all seven *Delftia* strains, demonstrating complete conservation of this secondary metabolite pathway. Each gene in the gene cluster is represented by an arrow. Conservative gene sequences among different gene clusters are linked by different colored segments, and the colors are classified according to the locus tags from the official MIBiG record. The genomic coordinates below the gene cluster represent the location on the genome.

By assembling second- and third-generation sequencing data, a complete genome sequence of 6,294,714 bp was finally obtained, with a G + C content of 67%. In total, 5,679 protein-coding genes of *D. lacustris* ZWP15 were annotated in the GO database (Fig. 2A).

For comparative analysis, six additional *D. lacustris* genomes were obtained from the NCBI, and the genomic sizes, GC content, gene number, CDS features, and accession numbers of all seven strains are presented in Table S2. Orthologous gene analysis identified 4,310 conserved protein families across all seven strains (Fig. 2B). The number of unique gene families varied greatly among the analyzed strains, with *D. lacustris* ZWP15 harboring only 4 compared to 26 in *D. tsuruhatensis* ULwDis3. The number of common gene families among ZWP15 and other *Delftia* strains ranges from 6 to 32. Importantly, we identified five gene families that are conserved across all three *D*. *lacustris* strains, as well as 54 gene families that are exclusively shared between the two *D*. *acidovorans* strains.

Secondary metabolites produced by biocontrol bacteria play a pivotal role in plant disease suppression. Genomic analysis revealed 10 distinct biosynthetic gene clusters across the seven *Delftia* strains, encoding pathways for Delftibactin A, Ripp-like peptides, Terpenes, Resorcinol, APE Vf, Pyxidicycline, Mycosubtilin, Thaxteramide, Gamexpeptide, and Arylpolyene (Table 2). Among them, Delftibactin A was conserved in all strains, exhibiting 100% sequence similarity. In strain ZWP15, the Delftibactin A biosynthetic cluster comprises 20 contiguous genes (Fig. 2C). Comparative analysis demonstrated high conservation of this gene cluster organization across all seven genomes, suggesting its essential functional role.

**TABLE 2:** Predicted secondary metabolite biosynthetic gene clusters in seven *Delftia* spp. based on antiSMASH analysis^*a*^^,^^*b*^

Cluster	*D. lacustris*			*D. acidovorans*		*D. tsuruhatenis*	*D. deserti*	Similarity
	ZWP15	DSM 21246	HQS1	RAY 209	SPH-1	ULwDis3	KCTC 42377	
Delftibactin A	+	+	+	+	+	+	+	100%
Ripp-like	+	+	+	+	+	+	+	/
Terpene	+	+	+	+	+	+	+	/
Resorcinol	+	+	+	+	+	+	+	/
Ape Vf	−	+	−	−	−		−	40%
Pyxidicycline	−	−	−	−	−	+	−	10%
Mycosubtilin	−	−	−	−	−		+	30%
Thaxteramide	−	−	−	−	−		+	11%
Gamexpeptide	−	−	−	−	−		+	16%
Arylpolyene						−	+	25%

^*a*^ Similarity refers to the percentage of genes in this gene cluster that are similar to the genes in previously reported gene clusters. Data are all based on antiSMASH analysis. “/” indicates that the sequence of this gene cluster have not been reported.

^*b*^ “+” indicates that the gene cluster exists in the corresponding strain, while “−” indicates its absence.

### Biocontrol by *Delftia lacustris* ZWP15 depends on Delftibactin A biosynthetic gene cluster

**Fig 3:**
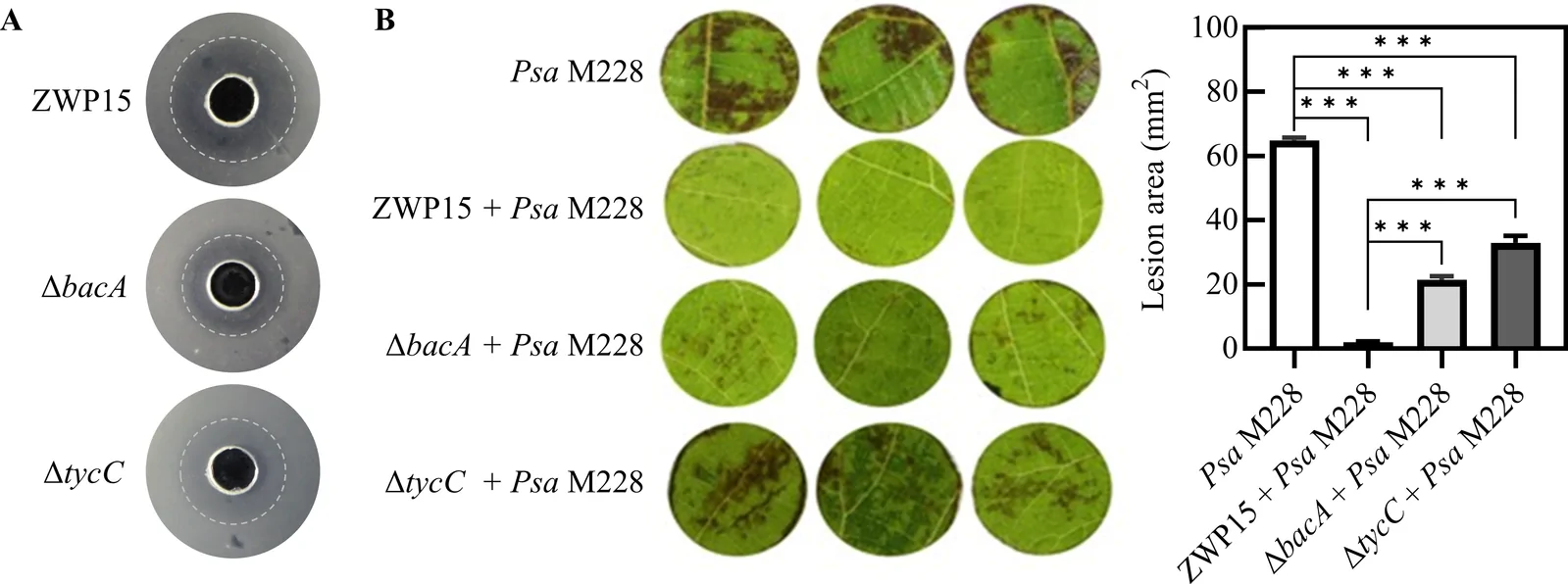
Functional validation of Delftibactin A in the biocontrol activity of *D. lacustris* ZWP15. (**A**) Antagonistic activity against *Psa* on LB agar plates. Wild-type ZWP15 and mutants (Δ*bacA* and Δ*tycC*) were evaluated using a dual-culture inhibition assay. (**B**) Disease suppression efficacy on kiwifruit leaves inoculated with *Psa*. Lesion development was quantified following treatment with wild-type ZWP15 or mutant strains. Data represent mean of three biological replicates, with asterisks indicating significant differences from wild-type control (***P < 0.001, Student’s *t*-test).

To determine the contribution of Delftibactin A in the antagonistic activity of *D. lacustris* ZWP15, we assessed the ability of mutants lacking the *tycC* or *bacA* core genes of the cluster to synthesize Delftibactin A (Fig. 1C). Phenotypic analysis revealed that the Δ*bacA* and Δ*tycC* mutants exhibited significantly reduced antagonistic activity, with inhibition rates decreasing by 65.77% and 60.93%, respectively, compared to the wild-type strain (Fig. 3A). Consistent with these findings, detached leaf assays demonstrated that disease suppression efficacy was impaired in both mutants, with Δ*bacA* showing a 67.51% reduction and Δ*tycC* showing a 42.96% reduction in protective activity (Fig. 3B). These results provide direct genetic evidence that Delftibactin A is a key bioactive metabolite contributing to the biocontrol capacity of *D. lacustris* ZWP15.

### *D. lacustris* ZWP15 impair virulence-related traits in *Psa* through Delftibactin A-dependent mechanisms

**Fig 4:**
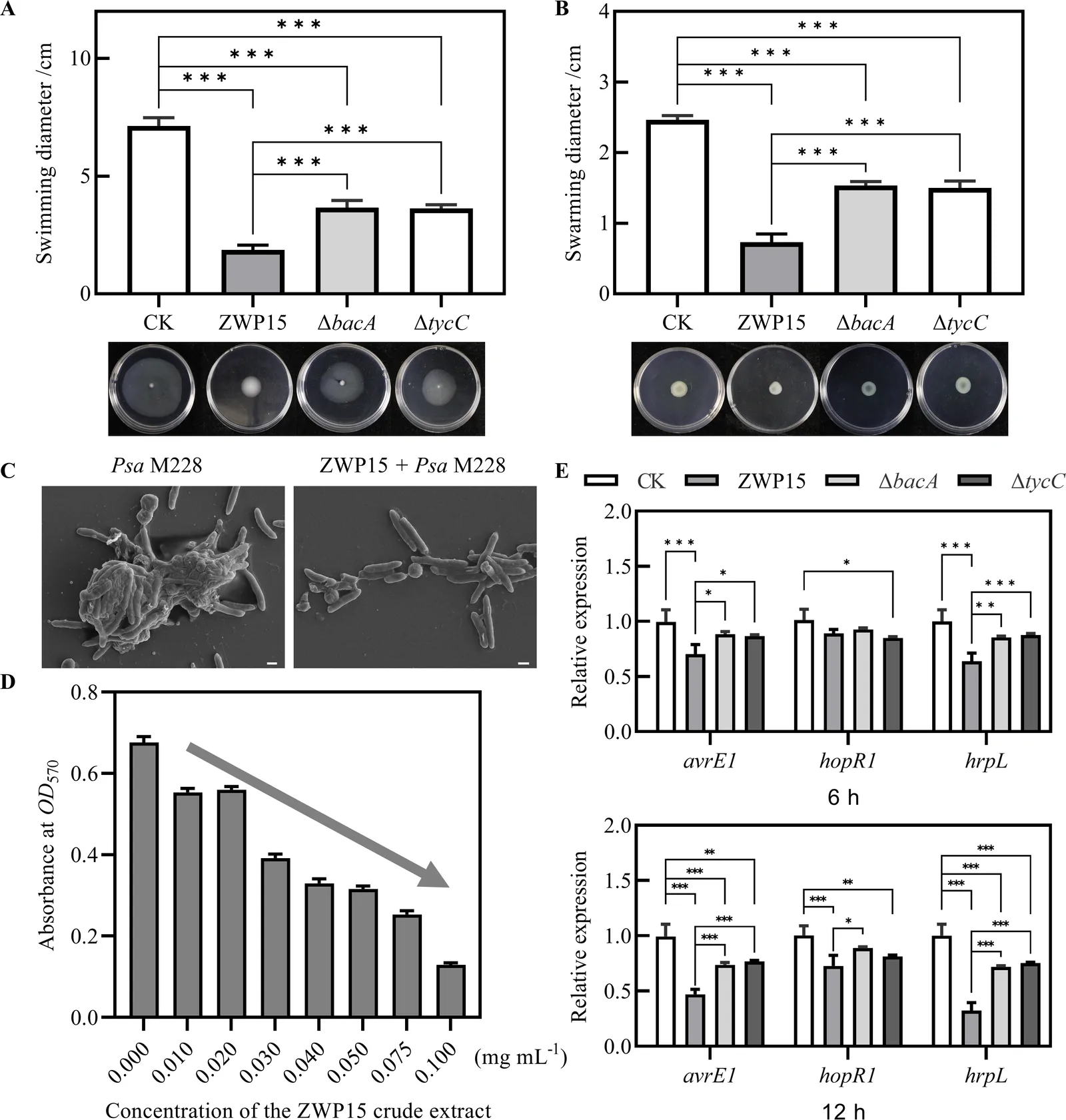
The crude extract affects the motility, biofilm formation, and expression of T3SS pathogenicity-related genes in *Psa*. (**A**) The swimming ability and (**B**) the swarming ability of *Psa* were reduced after treatment with *D. lacustris* ZWP15 crude extract. (**C**) SEM revealing structural disruption of *Psa* biofilms after extract treatment. Bar, 1 μm. (**D**) Biofilm biomass quantification using crystal violet staining. (**E**) RT-qPCR analysis of T3SS regulatory genes (*avrE1*, *hopR1*, and *hrpL*) showing transcript suppression by wild-type versus mutant extracts. Data represent the mean of three biological replicates, with asterisks indicating significant differences from wild-type control (***P < 0.001, **P < 0.002, and *P < 0.033, Student’s *t*-test).

To characterize the antimicrobial activity of *D. lacustris* ZWP15 metabolites, we examined the effects of crude extracts from wild-type and mutant strains (Δ*bacA* and Δ*tycC*) on key virulence determinants of *Psa*. Comparative analysis revealed significant differences in biological activity between extracts (Fig. 4).

The wild-type extract profoundly inhibited *Psa* motility, reducing swimming and swarming capacities by 73.77% and 70.32%, respectively (Fig. 4A and B). In contrast, extracts from Δ*bacA a*nd Δ*tycC* mutants showed attenuated effects, decreasing motility by only 38.06%–52.73% (swimming) and 39.27%–49.13% (swarming). Biofilm formation assays demonstrated complete disruption of extracellular matrix architecture by wild-type extract, while mutant extracts exhibited partial inhibition (Fig. 4C and D).

Transcriptional analysis indicated that different crude extracts exhibited significant variations in their suppression of key virulence genes associated with the T3SS in *Psa*. Wild-type extract strongly downregulated *avrE1*, *hopR1*, and *hrpL* expression (by 25% at 6 h and 49% at 12 h), whereas mutant extracts only inhibited 11% at 6 h and 22% at 12 h (Fig. 4E). Compared with the wild type, the inhibition rate of the mutant extract was even reduced by half. These results demonstrate that Delftibactin A contributes substantially to the extract’s ability to disrupt bacterial virulence mechanisms.

### *D. lacustris* ZWP15 mitigate *Psa*-induced host tissue damage through Delftibactin A-dependent mechanisms

**Fig 5:**
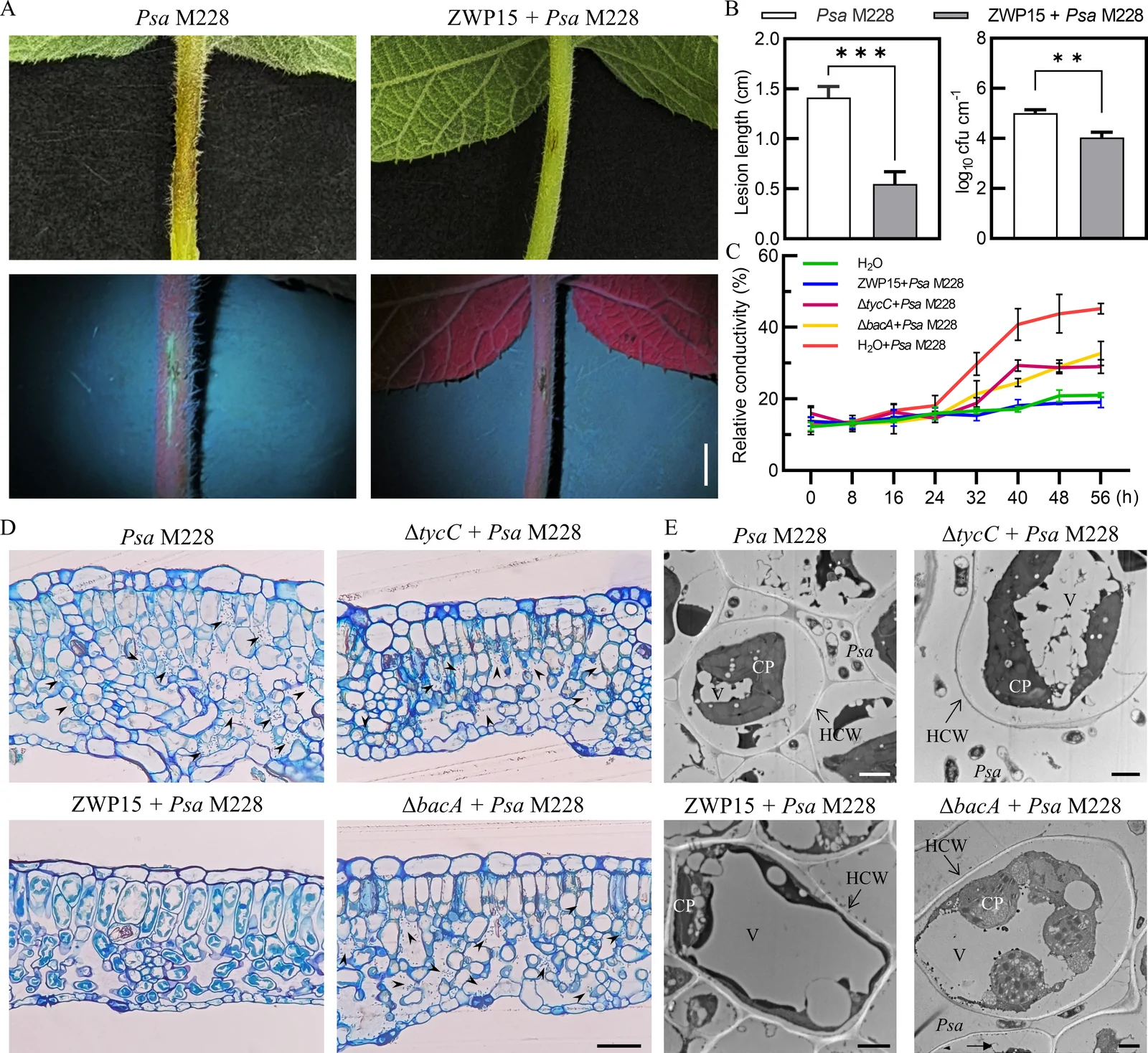
Electron microscopic observation of the inhibitory effect of crude extract of *D. lacustris* ZWP15 on *Psa*. (**A**) The inhibitory effect of crude extract of *D. lacustris* ZWP15 on the expansion ability of *Psa* M228 in kiwifruit leaves. Bar, 0.5 cm. (**B**) The bacterial content of the 1 cm long leaf stalk (**A**) above the vaccination site. (**C**) Relative conductivity of plant leaves inoculated with water, *Psa* M228, and the crude extract of *D. lacustris* ZWP15 (***P < 0.001 and **P < 0.002, Student’s *t*-test). (**D**) Histological observation of the host kiwifruit leaves inoculated with *Psa* M228, with or without pretreatment using the crude extracts from *D. lacustris* ZWP15, Δ*tycC,* or Δ*bacA*. UE, upper epidermis of the leaf; PT, palisade tissue; ST, spongy tissue; and LE, lower epidermis of the leaf. The arrows indicate *Pseudomonas syringae* pv. *actinidiae*. Bar, 30 μm. (**E**) Ultrastructure observation of the host kiwifruit leaves inoculated with *Psa* M228, with or without pretreatment using the crude extract from *D. lacustris* ZWP15. CP, chloroplast; V, vacuole; and HCW, host cell wall. Bar, 2 μm.

The inhibition of the crude extract on the expansion of *Psa* M228 was tested. After 3 days of inoculation, the pathogen migration distance in the control group reached 1.43 cm, while that in the treatment group was only 0.51 cm (Fig. 5A). Meanwhile, the colonization amount of *Psa* M228-GFP in the kiwifruit leaf vein tissue of the control group was 1.152 × 10^5^ CFU/cm, while after pretreatment with the crude extract of ZWP15, the colonization amount of *Psa* M228-GFP was only 1.6 × 10^4^ CFU/cm (Fig. 5B; Fig. S4). The crude extract of ZWP15 significantly inhibited the expansion ability of the pathogen in the host.

The relative conductivity of the negative control group did not change significantly, increasing only from approximately 12% to 20% throughout 0–56 h (Fig. 5C). After the *Psa* M228 infection, the relative conductivity of the host cell wall significantly increased from 12% to 45% over time after inoculation with *Psa* M228, indicating that *Psa* M228 infected the kiwi leaves, damaged the cell membranes, and caused a large leakage of intracellular electrolytes. When the Delftibactin A crude extract of *D. lacustris* ZWP15 was pretreated 1 day before *Psa* M228 infection, the relative conductivity also increased over time, but the increase was substantially lower than that observed with *Psa* M228 alone. Similarly, pretreatment with the Delftibactin A crude extract of Δ*tycC* and Δ*bacA* also led to an increase in relative conductivity, though to a lesser extent than *Psa* M228 alone. Specifically, Δ*tycC* pretreatment resulted in an increase from 15% to 27%, while Δ*bacA* pretreatment led to an increase from 15% to 33%. These results demonstrate that the Delftibactin A crude extract limits *Psa* colonization and preserves host cell integrity during infection.

The inhibition of the crude extract on the infectivity of *Psa* M228 to the host was also confirmed by the semithin section (Fig. 5D) and ultrathin section (Fig. 5E) of TEM. After inoculating the host kiwifruit leaves with *Psa* M228 for 3 days, a number of pathogens could be observed in the host tissue (Fig. 5D). However, in the treatment with the crude extract of *D. lacustris* ZWP15 prior to inoculation with *Psa* M228, only a small amount of pathogen colonization was observed in the host tissues at 3 days (Fig. 5D). Similarly, pretreatment with the Delftibactin A mutants Δ*tycC* and Δ*bacA* also resulted in reduced pathogen colonization compared with the Psa‑only control, though not as effectively as the crude extract (Fig. 5D).

The ultrathin section of TEM showed similar results. After inoculation with *Psa* M228, the healthy kiwi leaves suffered damage to the protoplasts, and the chloroplasts also underwent degradation (Fig. 5E). Treatment with the Delftibactin A crude extract of *D. lacustris* ZWP15 before inoculation with *Psa* M228 not only significantly reduced the colonization of *Psa* M228 but also alleviated the destruction of host cells by pathogens, and the host cells were relatively intact (Fig. 5E). Similarly, pretreatment with the Delftibactin A crude extract of mutants (Δ*tycC* and Δ*bacA*) also provided some protection, albeit to a lesser extent than the crude extract. These observations further confirm that Delftibactin A plays a key role in preserving host cell integrity during *Psa* infection.

## DISCUSSION

Kiwifruit (*Actinidia* spp.) is a globally important cash crop valued for its unique flavor and nutritional benefits (33). However, KBC has become a devastating disease, threatening production and causing substantial economic losses worldwide (3, 28, 34, 35). Current control of KBC still relies on the lack of sustainable strategies through the frequent use of harmful chemical pesticides, which are often ineffective, persistent in the environment, and cause serious pollution (6, 11, 36). Therefore, the search for novel, highly effective biocontrol strains remains critical. Currently, biocontrol of plant bacterial diseases is dominated by a few bacterial genera, with most reports focusing on *Bacillus* spp. and *Pseudomonas* spp., owing to their well-documented antagonistic mechanisms and ease of formulation (11, 37, 38). However, excessive reliance on a limited number of bacterial strains may lead to the emergence of drug resistance and may also limit the applicability of biological control strategies in different agricultural ecological regions. Expanding the biodiversity of biocontrol agents is therefore a pressing need. In this study, we report for the first time that *Delftia lacustris* ZWP15, isolated from kiwifruit rhizosphere soil, exhibits strong inhibition activity against KBC compared to commercial *Bacillus subtilis* formulations. Previous studies on *Delftia* spp. in plant disease management are scarce. *D. lacustris* PPO-1 has been shown to inhibit fungal pathogens in tomato (13), and *D. tsuruhatensis* MTQ3 from tobacco rhizosphere exhibits antagonistic activity against *Ralstonia solanacearum* and *Phytophthora nicotianae* (14). However, the potential of *Delftia* sp. as a biocontrol agent against bacterial phytopathogens, particularly in the context of KBC, has remained unexplored. Our study fills this gap by demonstrating that *D. lacustris* ZWP15 not only reduces *Psa* infection but also provides a genetic basis for its antagonistic mechanism through the Delftibactin A biosynthetic gene cluster. Nevertheless, the potential opportunistic pathogenicity of certain *Delftia* strains warrants careful biosafety assessment prior to field use.

In recent years, whole-genome sequencing has emerged as a powerful and efficient strategy to uncover the biosynthetic potential of microorganisms and guide the discovery of novel bioactive compounds. For instance, genome mining of *Pseudomonas chlororaphis* subsp. *aureofaciens* W9-1 led to the identification of the phenazine-1-carboxylic acid biosynthetic gene cluster, and subsequent functional studies demonstrated that PCA exerts its broad-spectrum antifungal effect by targeting isocitrate lyase (39). Similarly, *P. vancouverensis*, a bacterium with antifungal activity against crop pathogens, was found through genome mining to harbor a rich repertoire of BGCs encoding diverse secondary metabolites, including non-ribosomal peptides (NRPs), polyketides, and bacteriocins (40). These studies collectively demonstrate that genome-guided analysis can effectively predict the capacity of a microorganism to produce antimicrobial compounds, thereby accelerating target identification and functional validation. In our study, antiSMASH analysis of *D. lacustris* ZWP15 identified a biosynthetic gene cluster, the Delftibactin A cluster. This finding validates genome mining as a crucial first step in understanding the biocontrol potential of ZWP15 and justifies our subsequent focus on the Delftibactin A cluster.

Comparative genomics provides a robust framework to differentiate core and accessory genomic elements across related strains and is particularly effective for pinpointing genetic determinants underlying strain-specific phenotypes. This approach has been successfully employed to link genotype with biocontrol phenotype. For example, comparative genomic analysis of nine potato-associated *Pseudomonas* isolates with differing antagonistic potential against *Phytophthora infestans* revealed distinct arsenals of antimicrobial compounds, including phenazine, hydrogen cyanide, and various siderophores (41). EggNOG functional annotation of the ZWP15 genome revealed that genes with unknown function accounted for 27.05% of the total (Fig. S5). Therefore, to systematically mine the key bioactive determinants in ZWP15, we conducted comparative genomic analysis with five additional *Delftia* strains representing both major phylogenomic lineages (16). The results revealed that, despite extensive genomic plasticity and diversity across the genus, the Delftibactin A biosynthetic gene cluster stands out as a remarkably conserved element. This striking contrast implies that the *Delftia* sp. may achieve dual advantages in ecological niche occupation and pathogen suppression.

Delftibactin A was originally identified in *D. acidovorans* SPH-1 as a NRP involved in gold detoxification and biomineralization (42). Subsequent studies have demonstrated its broad-spectrum antimicrobial activity against both gram-positive multidrug-resistant bacteria, such as methicillin-resistant *Staphylococcus aureus* and vancomycin-resistant *Enterococcus*, as well as gram-negative pathogens, including *Acinetobacter baumannii* and *Klebsiella pneumoniae* (15). In the present study, we further uncover novel functions of Delftibactin A. Our results show that the crude extract Delftibactin A not only directly inhibits *Psa* growth but also suppresses key virulence traits of the pathogen, including swimming and swarming motility, biofilm formation, and the expression of T3SS-related genes (*avrE1*, *hopR1*, and *hrpL*). Moreover, treatment with the crude extract alleviated *Psa* infection and subsequent host cell destruction in kiwi leaves. Consistent with these observations is the fact that knocking out the key genes (Δ*tycC* and Δ*bacA*) in the Delftibactin A cluster led to a significant reduction in the biocontrol effect, confirming that Delftibactin A is the main contributing factor to the antagonistic activity of ZWP15, although it is not the sole determinant. Given that the crude extract was always used in this study, we acknowledge a limitation that we were unable to obtain pure Delftibactin A; therefore, the possibility that other metabolites in the crude extract may also contribute to the observed phenotypes cannot be fully excluded. Future work will focus on high-performance liquid chromatography-mass spectrometry-guided fractionation and purification to isolate pure Delftibactin A, followed by rigorous structural characterization and validation of its individual biological activities.

Genome annotation of *D. lacustris* ZWP15 revealed that genes with unknown function constitute a substantial proportion of its genome (approximately 27.05%). This observation strongly suggests that ZWP15 harbors a reservoir of previously unknown functional genes and biosynthetic pathways awaiting discovery. This redundancy in antagonistic mechanisms is not uncommon among potent biocontrol bacteria; for instance, superior *Bacillus* strains often harbor multiple biosynthetic gene clusters whose effects are additive or synergistic (43, 44). The presence of such parallel mechanisms may confer enhanced robustness and ecological competitiveness under fluctuating environmental conditions (45). In our future studies, we will systematically study the other predicted BGCs in ZWP15 through targeted gene knockouts and heterologous expression to understand the full set of weapons ZWP15 uses against *Psa*. Furthermore, given that Delftibactin A appears to act both as a direct antimicrobial agent and as a modulator of host-pathogen interactions, unraveling its complete mode of action, particularly its effects on plant immune responses, will be a priority. Elucidating these mechanisms will not only deepen our understanding of how *Delftia* spp. function as biocontrol agents but also provide a theoretical foundation for the rational design of broad-spectrum biocontrol formulations and the engineering of optimized strains with enhanced field efficacy.

## Data Availability

The genome sequence reported in this paper has been deposited in the NCBI database (https://www.ncbi.nlm.nih.gov/datasets/genome/GCF_041893715.1/).

## SUPPLEMENTAL MATERIAL

Supplemental material spectrum.00313-26-s0001.docx Table S1 and S2; Figures S1 to S5.

ASM does not own the copyrights to Supplemental Material that may be linked to, or accessed through, an article. The authors have granted ASM a non-exclusive, world-wide license to publish the Supplemental Material files. Please contact the corresponding author directly for reuse.
