# Supplementary material for: A conserved Delftibactin A biosynthetic gene cluster plays an important role in the biocontrol of kiwifruit bacterial canker by Delftia lacustris ZWP15
Source: Microbiol Spectr. 2026 May 29;14(7):e00313-26. doi: 10.1128/spectrum.00313-26 (PMC13339810; doi:10.1128/spectrum.00313-26)
Supplement: Supplemental material — Table S1 and S2; Figures S1 to S5. [file spectrum.00313-26-s0001.docx]

**Supporting Information**

Article title: **A conserved Delftibactin A biosynthetic gene cluster plays an important role for biocontrol of kiwifruit bacterial canker by *Delftia lacustris* ZWP15**

Authors: Jing Huang, Weipeng He, Yifei Liang, Xuyan Wu, Ruolan Yang, Xinying Liu, Zimeng Wang, Nana Wang^*^, Lili Huang^*^

The following Supporting Information is available for this article:

Table S1 Primers used in this study.

| Name | Sequence (5’-3’) | purpose |
| --- | --- | --- |
| *tycC*-1F | CTATGACATGATTACGAATTCAGACCTTCCAGCGCTTC | Δ*tycC* construct |
| *tycC*-2R | GTGGTTTCGGAGGAAGAAGACACAGCTGACATGCTGAGTGGA |  |
| *tycC*-3F | TGTCTTCTTCCTCCGAAACCAC |  |
| *tycC*-4R | ACGACGGCCAGTGCCAAGCTTCTATGTGGAAGCCCATGGCA |  |
| *bacA*-1F | CTATGACATGATTACGAATTCCTTGACGAAGGACTGCGC | Δ*bacA* construct |
| *bacA*-2R | AGACAAGGTGTAACCCGGTTCCTTTCTCGAAACTCTGGGAG |  |
| *bacA*-3F | ACCGGGTTACACCTTGTCT |  |
| *bacA*-4R | ACGACGGCCAGTGCCAAGCTTGGTGATCTTTGGCTCGGTCTC |  |
| *tycC*-F | CAGGCAGACACGCTCTTCAC | Δ*tycC* detection |
| *tycC*-R | ACCAGCACCAGGGACACGC |  |
| *bacA*-F | GGTGGCGTTGACCTGCGA | Δ*bacA* detection |
| *bacA*-R | TCCGTCTTGGTGATCTCCCG |  |

Table S2 General genome features of the seven *Delftia* sp.

| Strains | Genome size (Mb) | GC (%) | Gene number | CDS features | Accession number |
| --- | --- | --- | --- | --- | --- |
| ZWP15 | 6.3 | 67 | 5,679 | 5,541 | SAMN43478800 |
| DSM 21246 | 7.3 | 66.5 | 6,661 | 6,489 | SAMN38892012 |
| HQS1 | 7 | 66.5 | 6,434 | 6260 | SAMD00407106 |
| RAY209 | 6.5 | 66.5 | 5,929 | 5,769 | SAMN07410697 |
| SPH-1 | 6.8 | 66.5 | 6,275 | 6,138 | SAMN00623041 |
| ULwDis3 | 6.9 | 66.5 | 6,477 | 6,311 | SAMN33417407 |
| KCTC 42377 | 7.1 | 66.5 | 6,707 | 6,498 | SAMN43282975 |


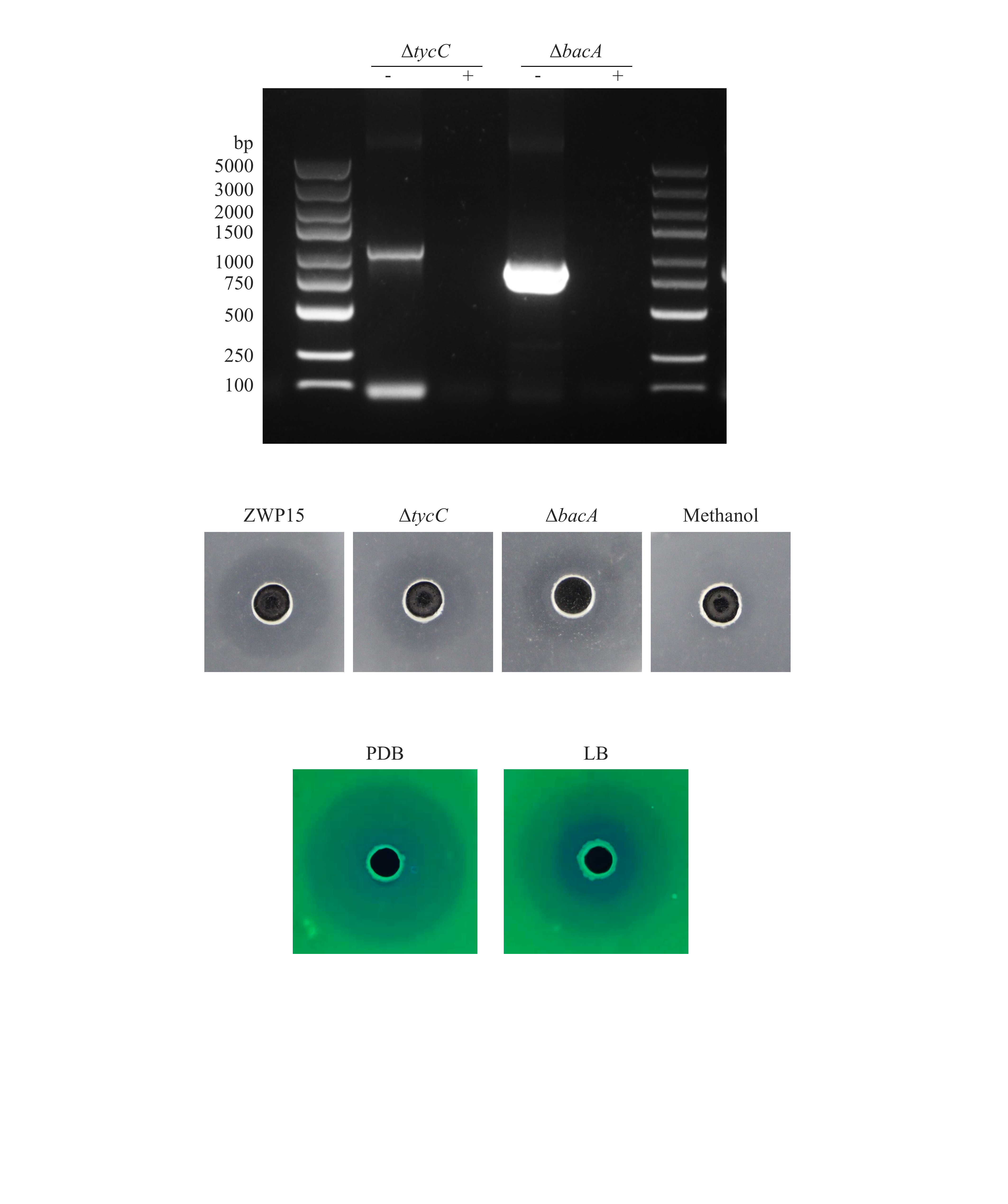


**Fig. S1 Antagonistic activity of the fermentation broths obtained from ZWP15 cultured in Potato Dextrose Broth (PDB) and LB liquid media.**


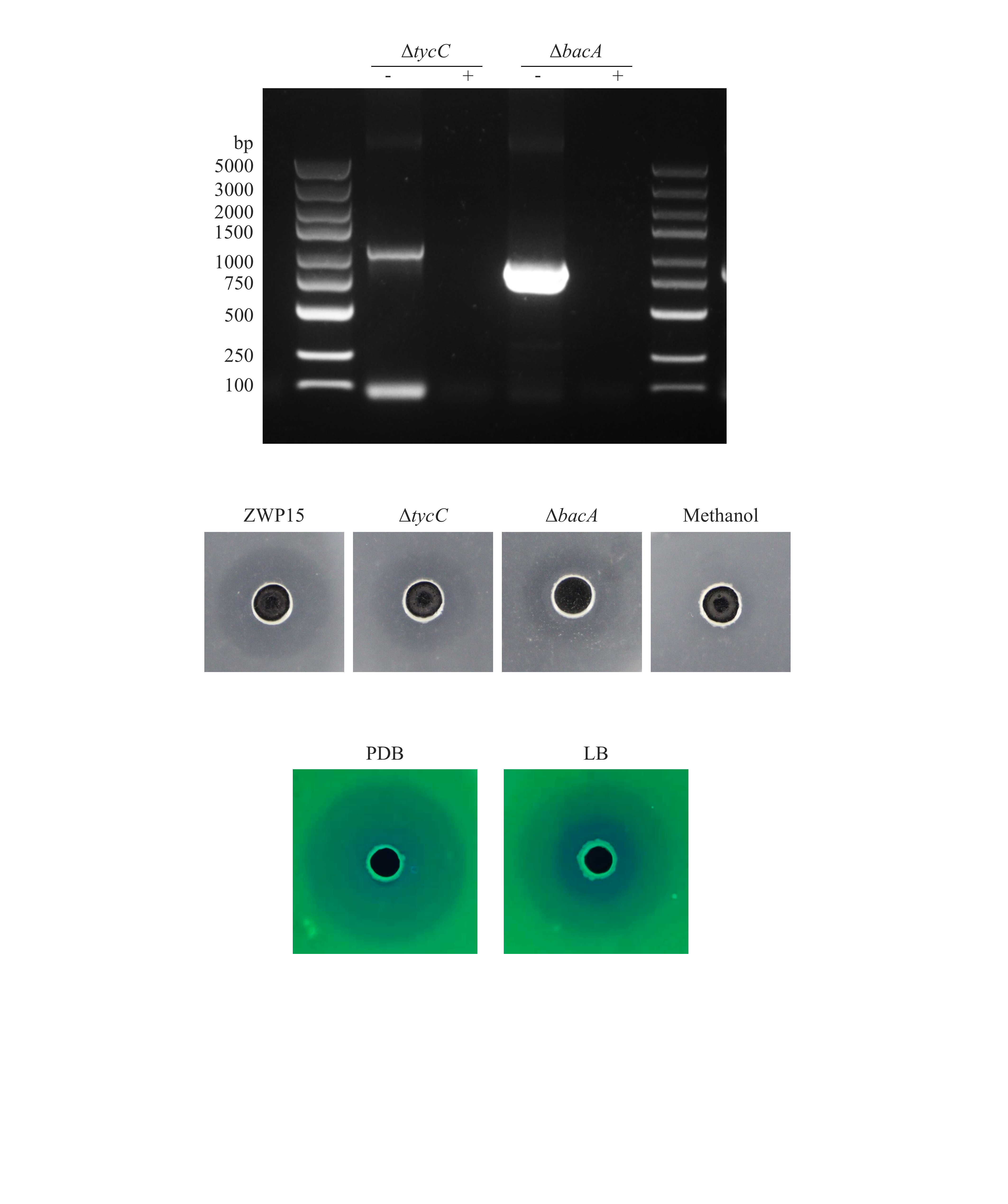


**Fig. S2 Construction of gene deletion mutants in ZWP15 (Δ*tycC* and Δ*bacA*).** The deletion mutants (Δ*tycC* and Δ*bacA*) were verified with primer pairs *tycC*-F/R, and *bacA*-F/R, respectively.


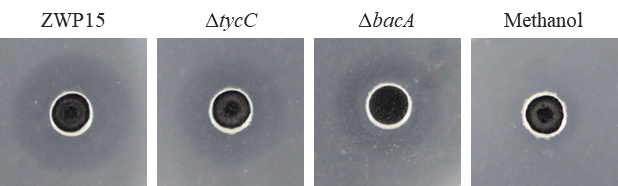


**Fig. S3 Antagonistic activity of Delftibactin A crude extract against *Psa*.** The antagonistic activity of the crude extracts was evaluated, and the Methanol was used as the negative control.


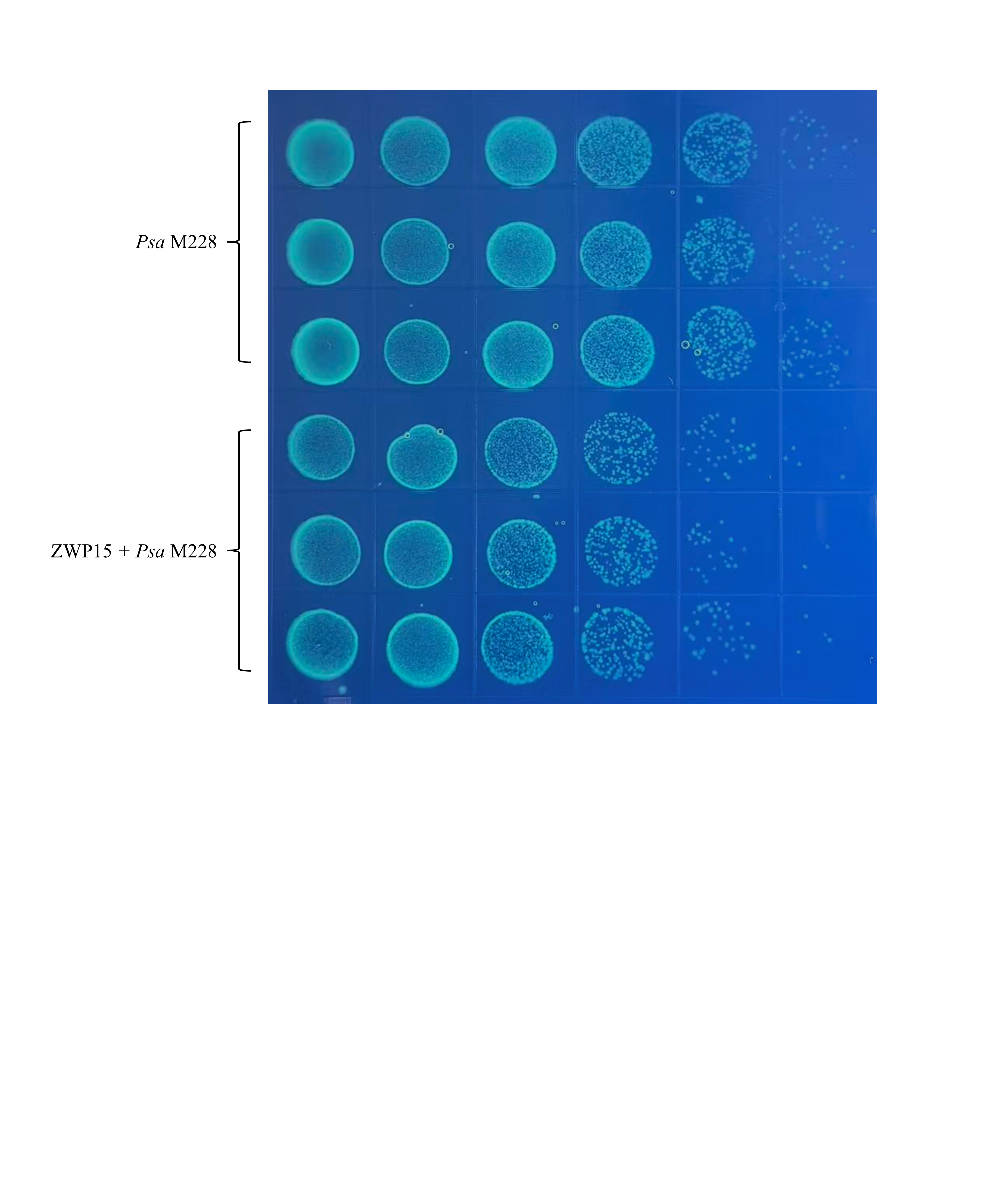


**Fig. S4 The inhibitory effect of Delftibactin-A crude extract on the bacterial content of *Psa* M228-GFP in kiwifruit leaves.** The bacterial content of the 1 cm long leaf stalk (Fig. 5A, B) above the vaccination site.


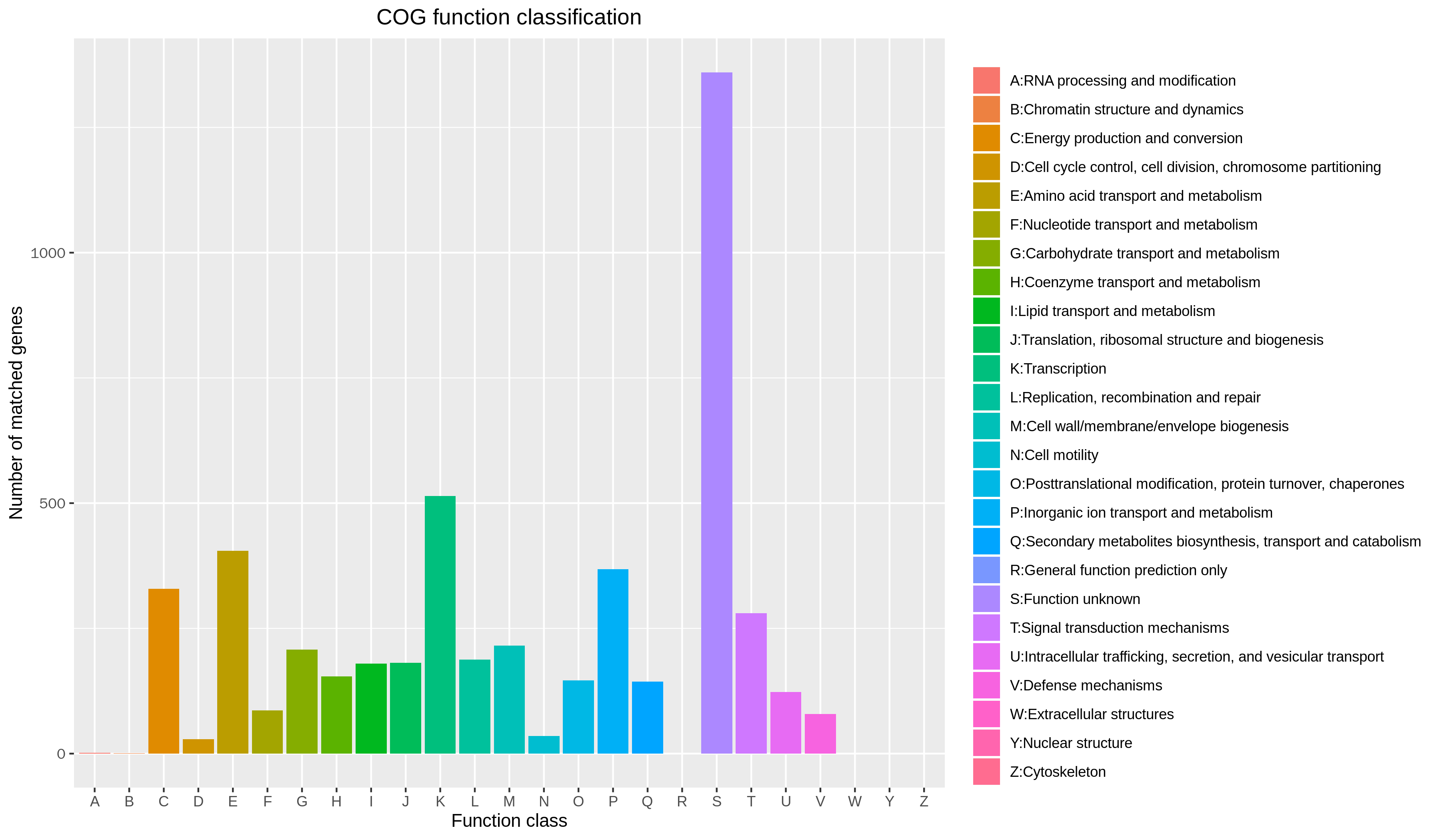


**Fig. S5 EggNOG annotation of the protein-coding gene of ZWP15.**
